# Rapid and easy detection of low-level resistance to vancomycin in methicillin-resistant *Staphylococcus aureus* by matrix-assisted laser desorption ionization time-of-flight mass spectrometry

**DOI:** 10.1371/journal.pone.0194212

**Published:** 2018-03-09

**Authors:** Kota Asakura, Takuya Azechi, Hiroshi Sasano, Hidehito Matsui, Hideaki Hanaki, Motoyasu Miyazaki, Tohru Takata, Miwa Sekine, Tomoiku Takaku, Tomonori Ochiai, Norio Komatsu, Keigo Shibayama, Yuki Katayama, Koji Yahara

**Affiliations:** 1 Department of Pharmacy, Juntendo University Hospital, Tokyo, Japan; 2 Infection Control Research Center, Kitasato Institute for Life Science, Kitasato University, Tokyo, Japan; 3 Department of Pharmacy, Fukuoka University Chikushi Hospital, Fukuoka, Japan; 4 Department of Infection Control, Fukuoka University Hospital, Fukuoka, Japan; 5 Department of Microbiology, Faculty of Medicine, Juntendo University, Tokyo, Japan; 6 Division of Hematology, Department of Internal Medicine, Juntendo University, Tokyo, Japan; 7 Department of Bacteriology II, National Institute of Infectious Diseases, Tokyo, Japan; 8 Antimicrobial Resistance Research Center, National Institute of Infectious Diseases, Tokyo, Japan; Pusan National University, REPUBLIC OF KOREA

## Abstract

Vancomycin-intermediately resistant *Staphylococcus aureus* (VISA) and heterogeneous VISA (hVISA) are associated with treatment failure. hVISA contains only a subpopulation of cells with increased minimal inhibitory concentrations, and its detection is problematic because it is classified as vancomycin-susceptible by standard susceptibility testing and the gold-standard method for its detection is impractical in clinical microbiology laboratories. Recently, a research group developed a machine-learning classifier to distinguish VISA and hVISA from vancomycin-susceptible *S*. *aureus* (VSSA) according to matrix-assisted laser desorption ionization time-of-flight mass spectrometry (MALDI-TOF MS) data. Nonetheless, the sensitivity of hVISA classification was found to be 76%, and the program was not completely automated with a graphical user interface. Here, we developed a more accurate machine-learning classifier for discrimination of hVISA from VSSA and VISA among MRSA isolates in Japanese hospitals by means of MALDI-TOF MS data. The classifier showed 99% sensitivity of hVISA classification. Furthermore, we clarified the procedures for preparing samples and obtaining MALDI-TOF MS data and developed all-in-one software, hVISA Classifier, with a graphical user interface that automates the classification and is easy for medical workers to use; it is publicly available at https://github.com/bioprojects/hVISAclassifier. This system is useful and practical for screening MRSA isolates for the hVISA phenotype in clinical microbiology laboratories and thus should improve treatment of MRSA infections.

## Introduction

Vancomycin has been the first-line drug for the treatment of methicillin-resistant *S*. *aureus* (MRSA) infections [1] prevalent worldwide [2]. Vancomycin-nonsusceptible MRSA infections are associated with greater rates of clinical treatment failure in comparison with vancomycin-susceptible *S*. *aureus* (VSSA) infections [3, 4]. Vancomycin-nonsusceptible MRSA can be classified into vancomycin-resistant *S*. *aureus*, vancomycin-intermediately resistant *S*. *aureus* (VISA), and heterogeneous VISA (hVISA), in which only a subpopulation of cells has minimal inhibitory concentrations (MICs) within the range indicative of intermediate resistance [5]. The hVISA phenotype is thought to be an intermediate step in the evolution of VSSA to VISA for the development of resistance [6–9]. Treatment failures associated with hVISA have been reported [10, 11]. Moreover, the detection of hVISA is known to be problematic because this phenotype is not detected by standard susceptibility testing; hVISA is classified as susceptible by the CLSI (Clinical & Laboratory Standards Institute) standards (MIC of vancomycin ≤2 μg/ml) [12].

hVISA has a subpopulation of cells resistant to 4 μg/ml vancomycin at a frequency ≥ 10^−7^ [5, 13]. Although the population analysis profile-area under the curve ratio (PAP-AUC) is the gold standard of hVISA detection, it is time-consuming, expensive, and impractical for implementation in clinical microbiology laboratories [5, 14, 15]. As an alternative, recently, a research group developed a machine-learning classifier to distinguish VISA and hVISA from VSSA on the basis of matrix-assisted laser desorption ionization time-of-flight mass spectrometry (MALDI-TOF MS) data of MRSA isolates consisting of VISA, hVISA, and VSSA [15]. MALDI-TOF MS is becoming popular in clinical laboratories because it enables detection of bacteria at a low running cost. That study showed an overall classification accuracy of 89%.

Nevertheless, classification of VISA is much easier than that of hVISA. The machine-learning classifier in that study (which involves MALDI-TOF MS data) showed 100% sensitivity of VISA classification, whereas 76% sensitivity of hVISA classification. This difference in sensitivity was expected because unlike VISA, hVISA cannot be detected by standard susceptibility testing. In addition, not all source codes were made public to completely automate the classification, and no graphical user interface was developed. In the present study, we focused on the more difficult objective of discriminating hVISA from VSSA and VISA among MRSA isolates in Japanese hospitals; we developed a more accurate machine-learning classifier using MALDI-TOF MS data. In addition, we clarified the procedures for preparing samples and obtaining MALDI-TOF MS data. Furthermore, we implemented the proposed approach as all-in-one software with a graphical user interface that automates the classification and is easy for medical workers to use.

We demonstrated that the newly developed discrimination process has sensitivity of 99% and specificity of 88% and we made the software publicly available.

## Materials and methods

### Bacterial strains and culture conditions

The bacterial strains used in this study were routinely grown in the brain heart infusion (BHI) agar medium (Eiken Chemical Co., Ltd. Tochigi, Japan) at 37°C. We analyzed 127 hospital-acquired MRSA (HA-MRSA) isolates that were collected from 1987 to 2011 at 13 hospitals in Japan [16–18] (S1 Table). Bacterial strain typing was performed for some isolates for the identification of SCC*mec* and *agr* elements. We also used hVISA clinical strain Mu3 [5] and VISA clinical strain Mu50 [7] as a control, resulting in 129 isolates in total. Among them, we analyzed 32 VSSA, 65 hVISA, and 32 VISA strains, and sometimes picked multiple colonies from the same hVISA strain to examine potential phenotypic differences among the colonies. To be precise, there were 10, 11, 2, and 1 strains from which we picked 2, 3, 4, and 5 colonies per strain, respectively, which adds up to 171 colonies of VSSA, hVISA, or VISA (Table 1).

**Table 1:** Performance of the software for classification into VISA, hVISA and VSSA.

	Prediction		
True	VISA	hVISA	VSSA
VISA	32	0	0
hVISA	0	106	1
VSSA	0	4	28

### Identification of phenotypic expression of resistance to vancomycin

The value of MIC of vancomycin has been reported previously [16, 18]. In brief, MICs of antibiotics were determined by the micro broth dilution method according to CLSI guidelines [19]. To detect the hVISA phenotype in MRSA isolates, we determined the plating efficiency of each isolate on the agar medium containing 4 μg/ml vancomycin by population analysis, as described elsewhere [13]. hVISA status was defined as follows: isolates that grew on the agar medium containing 4 μg/ml vancomycin with a frequency ≥ 10^−7^ colonies, 72 hours after 10^8^ cells were inoculated [13].

### Sample preparation and MALDI-TOF MS

A single colony grown on BHI-agar was subjected to an extraction procedure with the ethanol–formic acid–acetonitrile mixture, according to the manufacturer’s recommendations (Bruker Daltonics, Nillerica, MA) [20] with some modifications. In brief, a 1-μl loopful of bacterial cells was resuspended in 300 μl of sterilized Milli-Q water, and 900 μl of 99% ethanol (WAKO Pure Chemical Industries, Ltd. Osaka, Japan) was added to the cells. After centrifugation at 20400 × *g* for 3 min, the cell suspension was incubated at 50°C for 5 min. Next, 50 μl of 70% formic acid (Wako) and 50 μl of acetonitrile (Wako) were added to the cell suspension. The components were thoroughly mixed, centrifuged at 20400 × *g* for 3 min, and the supernatant was then transferred to a fresh tube to serve as a sample. After that, 1 μl of a MALDI matrix (a saturated solution of α-cyano-4-hydroxycinnamic acid [Bruker Daltonics] in 50% acetonitrile [Wako] and 2.5% trifluoroacetic acid [Wako]) was applied to each 1-μl sample and dried. MALDI-TOF MS was performed on a MicroFlex mass spectrometer in the range of mass/charge (m/z) values of 1000 to 20,000 (Bruker Daltonics) using by MALDI Biotyper RTC or MALDI Biotyper Compass.

### Data processing, classification, and software implementation

Data processing was based on the functions available in the MALDIquant package [21] of the R software. MALDI-TOF MS data was imported by the importBrukerFlex function. The imported data were processed by functions transformIntensity, smoothIntensity, and removeBaseline. Peaks in the spectrum of each sample were detected via the detectPeaks function and were aligned among the samples by means of functions warpMassPeaks and binPeaks, resulting in a data matrix in which rows represented the samples and columns denoted the aligned peaks. A random forest machine-learning classifier was constructed from the matrix after variable selection; in this procedure, a combination of peaks yielding the smallest out-of-bag error rate was selected by an algorithm implemented in the varSelRF package [22]. This algorithm iteratively fitted random forests to the data, building a new forest after discarding a fraction of peaks with the smallest variable importance. The graphical user interface was built using the Shiny package (http://shiny.rstudio.com).

### Ethics statement

This study’s protocol was approved by the local institutional review board (IRB) of Juntendo University (approval number 15–218), Fukuoka University Hospital (approval number 15-12-18) and Kitasato University (approval number B16-171). The HA-MRSA isolates used in the present study were originally collected in the previous studies (16, 17, 18) with informed consent. All samples were fully de-identified before we accessed them.

## Results

### Accurate classification into hVISA and VSSA using MALDI-TOF MS data

The classification of strains via MALDI-TOF MS data was successful (correct) for 32 out of 32 VISA colonies, for 106 out of 107 hVISA colonies, and for 28 out of 32 VSSA colonies (Table 1).

Sensitivity of the method was found to be 99% (23% higher than that in the previous study [15]), whereas specificity is 88%. As an advance relative to the other study [15], the algorithm constructed a machine-learning classifier after automatically selecting a combination of peaks in the spectra that were most effective at classifying the samples (see Discussion in detail). Representative average spectra for VSSA, hVISA, and VISA are shown in S1 Fig. Sensitivity and specificity were evaluated by leave-one-out cross-validation, where the machine-learning classifier was constructed after serial removal of a sample from the dataset with subsequent prediction for the removed sample.

In the dataset, strain typing was performed on some samples for identification of SCC*mec* [23] and *agr* elements [24]. It was found that 1, 22, and 18 hVISA samples and 4, 12, and 2 VSSA samples were of the SCC*mec* type I, II, and IV, respectively. Besides, 5, 16, and 16 hVISA samples and 5, 11, and 1 VSSA samples were of the *agr* type I, II, and III, respectively. The classification of hVISA was accomplished with 99% sensitivity across the different SCC*mec* and *agr* types. Meanwhile, the minor misclassification of VSSA was not confined to a specific SCC*mec* or *agr* type because the 3 misclassified VSSA samples belonged to type I or II or lacked the requisite SCC*mec-* and *agr*-related typing information.

### Software and its graphical user interface

**Fig 1:**
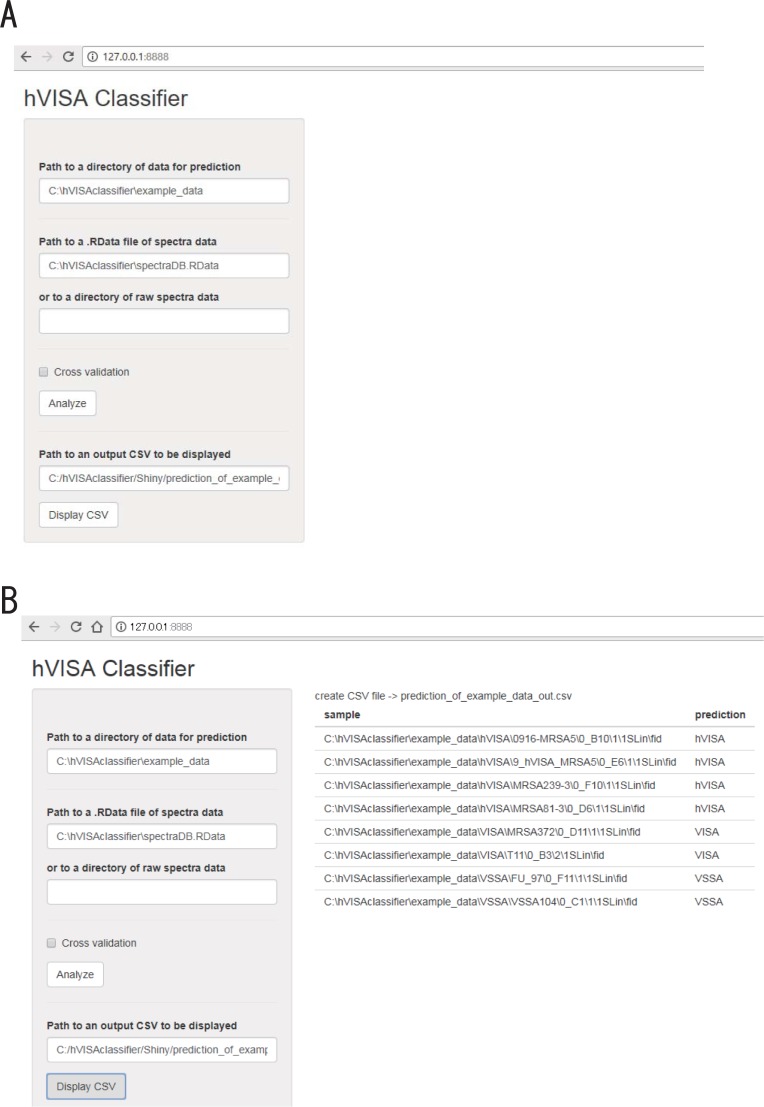
The graphical user interface of the classifier software. A software window is displayed in a web-browser. **(A) Use of the software.** The software interface can be easily used by specifying the path to a directory containing the MALDI-TOF MS data for each sample to be classified and by specifying a database file containing spectral data with a .RData extension (for example, the “example_data” directory and “spectraDB.RData” file are included in the software package) and by clicking on the “Analyze” button. **(B) Output.** After the computation is finished, an output CSV file is created. Its name and file path are automatically displayed in the browser window. Clicking on the “Display CSV” button yields predictions for each sample, which are displayed in the browser window.

To make the classification algorithm publicly available in an easy-to-use format, we developed software (called hVISA Classifier) with a graphical user interface; a screenshot of this software is presented in Fig 1.

Once the software is started, a simple window is displayed in a web browser. The spectral data of the VISA, hVISA and VSSA samples analyzed in this study are included in this software package as a database file. A user can employ it as a training dataset to construct a classifier or analyze their own raw MALDI-TOF MS data by specifying either of these in the program window (Fig 1A). Next, after specifying the raw MALDI-TOF MS data of samples to be classified, users can start the computation and view results in the browser window by clicking on the buttons (Fig 1B). The computation is quick, requiring a few minutes for 8 samples included in the package to be classified. This is a portable all-in-one software package that does not need the user to execute an installer or install any other additional program. The software package, including all source code, is downloadable at https://github.com/bioprojects/hVISAclassifier.

## Discussion

In the present study, we created a machine-learning classifier after automatically selecting a combination of peaks in the spectra that were most effective at classifying the samples; this approach is an improvement over the previous study [15] based on a standard support vector machine learning algorithm that was not capable of the automatic peak selection. The previous study only wrote that the peak selection was accomplished by forward stepwise multiple regression, but did not provide any more information including a source code to automate it. Furthermore, because the peaks in the spectra are expected to be correlated (i.e., presence of a peak can be associated with presence or absence of another peak), there is no theoretical justification to use multiple regression assuming independence among explanatory variables. On the other hand, peak selection in our procedure is clear by using a variable-selection algorithm for random forest machine-learning classifier implemented in the varSelRF package [22] and by making the source code public.

The effectiveness of the newly developed method was demonstrated by its sensitivity and specificity, which are 99% and 88%, respectively. The sensitivity was 23% higher than that in the previous study [15], whereas the specificity (28/32 = 88%) was almost the same as that (34/38 = 89%). It suggests that the VSSA training data in both the present and previous studies are not large or systematic enough. Further studies are warranted to increase MALDI-TOF mass-spectral data of VSSA samples more systematically.

The combination of peaks selected by the algorithm as most effective at classifying the samples was as follows: 1434, 1460, 1519, 1789, 1839, 1974, 2049, 2242, 2345, 2894, 2936, 4306, 4334, 5438, 5873, 6614, 6745, 6847, 6922, 7061, 8119, 8919, and 9697. All the peaks showed statistically significant difference among VISA, hVISA, and VSSA (*p* < 10^−4^, Kruskal-Wallis test). However, we should keep in mind that the present study did not validate stability of the selected peaks, and they may vary if another dataset is used for constructing the classifier. Therefore, we did not examine the biological meaning of the selected peaks, and our software did not yield the peaks as an output. Further studies are warranted to validate stability of the peaks as well as the sensitivity and specificity using another independent dataset.

We compressed the MALDI-TOF mass-spectral data [of 107 hVISA, 32 VSSA, and 32 VISA colonies (Materials and Methods) collected from more than 10 Japanese hospitals and examined in the present study] into a database file. We included it in the software package so that users can directly apply it to classifying their own MRSA samples into VISA, hVISA and VSSA easily. To conduct the classification of MRSA samples in other countries, the database would not be enough to obtain sufficient sensitivity and specificity. In such a case, another database should be built from the MALDI-TOF MS data on VISA, hVISA and VSSA collected in that country. For this purpose, the graphical user interface of the software has another text box (“or to a directory of raw spectral data” in Fig 1). Users can specify a directory in which raw spectral data of each sample are stored. Within this directory, users can further choose to store data in the “VISA”, “hVISA” or “VSSA” subdirectories. The raw spectral data are imported into the software, compressed into another database file, and processed by the algorithm to construct a machine-learning classifier after selecting another combination of peaks that were most effective at classifying the samples. Currently, the software can import the spectral data of MALDI-TOF MS instruments made by Bruker.

For sample preparation for MALDI-TOF MS, a single colony grown on BHI agar or on its with vancomycin 4 μg/ml was picked for extraction with ethanol–formic acid–acetonitrile mixture, according to the manufacturer’s recommendation (Bruker Daltonics, Nillerica, MA) with some modifications (as described in Materials and Methods). Owing to the thick cell wall of vancomycin-nonsusceptible MRSA strains, other protein extraction methods were not effective. We expect that the procedures clarified in the present study will form the basis for MALDI-TOF MS testing for vancomycin-nonsusceptible MRSA.

Although there has been no survey regarding the number of clinical laboratories that currently screen isolates for the hVISA phenotype [25], several studies have shown the occurrence of hVISA in many countries [26–32]. For example, in an international cohort of patients with infective endocarditis among 8 countries, the hVISA phenotype was detected by the population analysis profiling in more than one-quarter (19 of 65) of MRSA isolates associated with infective endocarditis [28]. A study in a UK hospital revealed that the prevalence of hVISA among clinical MRSA isolates, as determined by means of a modified vancomycin population analysis profile (PAP), is more than 5% [33]. A recent study of blood-borne MRSA isolates in Japan from 2008 to 2011 indicates that the prevalence of hVISA in 14 Japanese hospitals, according to a screening method employed in our study, is 6.5% [18]. Overall, these studies showed a wide range of prevalence estimates, and this variation may be due to reflect the differences in hVISA definitions and testing across studies and laboratories. Historically, hVISA was defined using BHI agar to test vancomycin resistance levels [5], but it is unknown why, and there is no comparative study of the MICs obtained with BHI agar and Müller-Hinton (MH) agar [34] more generally used in hospitals. Further studies are warranted to investigate how to define hVISA to solve the differences in hVISA definitions and testing across studies and laboratories.

We expect our rapid and easy-to-use method based on MALDI-TOF MS data to improve detection and screening of MRSA isolates for the hVISA phenotype worldwide. This method will lead to improve estimates of prevalence as well as treatment of MRSA infections via avoidance of vancomycin therapy in the presence of the hVISA phenotype.

## Supporting information

S1 Fig Representative average spectra for VSSA, hVISA, and VISA. (PDF)

S1 Table HA-MRSA isolates collected from 1987 to 2011 in Japan. (XLSX)
