# Supplementary figures and images for: Rapid and easy detection of low-level resistance to vancomycin in methicillin-resistant Staphylococcus aureus by matrix-assisted laser desorption ionization time-of-flight mass spectrometry
Source: PLoS One. 2018 Mar 9;13(3):e0194212. doi: 10.1371/journal.pone.0194212 (PMC5844673; doi:10.1371/journal.pone.0194212)

**VSSA**

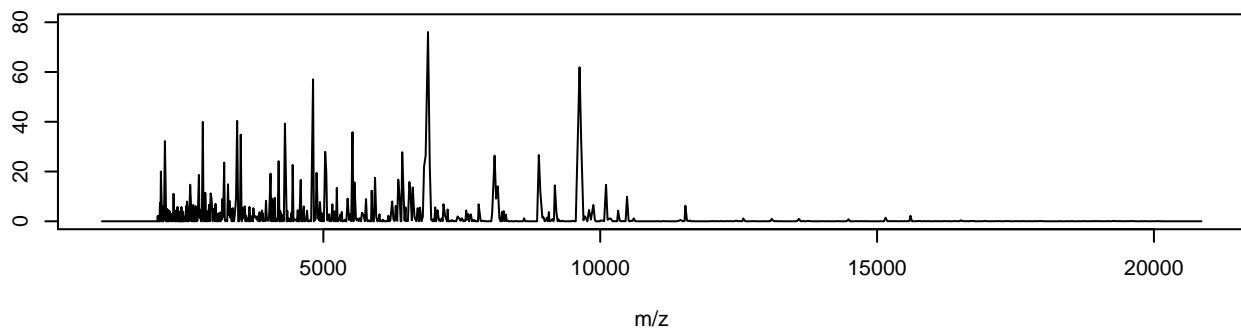

**hVISA**

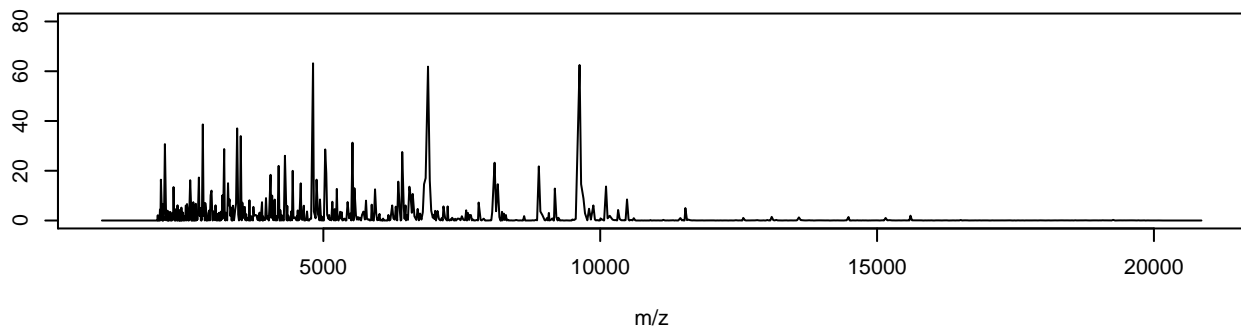

**VISA**

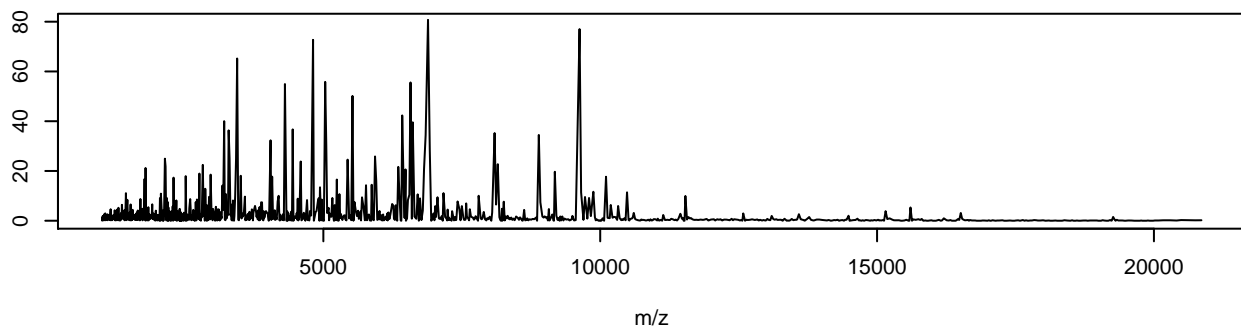

Supplement: S1 Fig — (PDF) [file pone.0194212.s001.pdf]
